# Outcomes of Zika virus infection during pregnancy: contributions to the debate on the efficiency of cohort studies

**DOI:** 10.1186/s12889-017-4915-2

**Published:** 2017-12-02

**Authors:** Elisabeth Carmen Duarte, Leila Posenato Garcia, Wildo Navegantes de Araújo, Maria P. Velez

**Affiliations:** 1 Medical School, University of Brasilia– UnB, Brasília, Brazil; 2 Núcleo de Medicina Tropical, Campus Universitário Darcy Ribeiro, Asa Norte, Caixa Postal 4517, Brasília, DF 70910-900 Brazil; 3 Institute of Applied Economic Research – IPEA, Brasília, Brazil; 4 Institute of Applied Economic Research – SBS Quadra 1, Bloco J, Ed. BNDES/Ipea, Brasília, DF 70076-900 Brazil; 5 Faculty of Ceilândia, University of Brasília – UnB, Brasília, Brazil; 6 School of Medicine – Department of Obstetrics and Gynaecology, Queen’s University, 99 University Ave, Kingston, ON K7L 3N6 Canada

**Keywords:** Zika virus infection, Cohort studies, Efficiency, Pregnancy outcome, Congenital abnormalities, Epidemiologic studies

## Abstract

**Background:**

Zika infection during pregnancy (ZIKVP) is known to be associated with adverse outcomes. Studies on this matter involve both rare outcomes and rare exposures and methodological choices are not straightforward. Cohort studies will surely offer more robust evidences, but their efficiency must be enhanced. We aim to contribute to the debate on sample selection strategies in cohort studies to assess outcomes associated with ZKVP.

**Main body of the abstract:**

A study can be statistically more efficient than another if its estimates are more accurate (precise and valid), even if the studies involve the same number of subjects. Sample size and specific design strategies can enhance or impair the statistical efficiency of a study, depending on how the subjects are distributed in subgroups pertinent to the analysis. In most ZIKVP cohort studies to date there is an a priori identification of the source population (pregnant women, regardless of their exposure status) which is then sampled or included in its entirety (census). Subsequently, the group of pregnant women is classified according to exposure (presence or absence of ZIKVP), respecting the exposed:unexposed ratio in the source population. We propose that the sample selection be done from the a priori identification of groups of pregnant women exposed and unexposed to ZIKVP. This method will allow for an oversampling (even 100%) of the pregnant women with ZKVP and a optimized sampling from the general population of pregnant women unexposed to ZIKVP, saving resources in the unexposed group and improving the expected number of incident cases (outcomes) overall.

**Conclusion:**

We hope that this proposal will broaden the methodological debate on the improvement of statistical power and protocol harmonization of cohort studies that aim to evaluate the association between Zika infection during pregnancy and outcomes for the offspring, as well as those with similar objectives.

## Background

Evidence to date indicates that infection by the Zika virus during pregnancy (ZKVP) is associated with the occurrence of microcephaly and other malformations of the central nervous system [1]. However, a large gap in knowledge still exists and further studies are needed to better understand this association. In this context experimental studies are ethically unviable, and analytical observational studies (cohort and case-control) are the only options available to test etiological and prognostic hypotheses [2].

Approaches of the case-control type are more efficient when studying rare outcomes, but have a high potential for selection and differential misclassification biases. Cohort designs are more efficient when studying rare exposures, with the advantage of minimizing some biases. However, Zika infection during pregnancy involves both rare outcomes and rare exposures, and therefore, the methodological choices are not straightforward. Cohort studies will surely offer more robust evidences, but its efficiency must be enhanced. The objective of this article is to contribute to the debate on sample selection strategies in cohort studies to assess outcomes associated with ZKVP.

## Discussion

### Efficiency of epidemiological studies

Rothman and Greenland (2008) define study efficiency as the relationship between the amount of information extracted from the data and the number of subjects recruited for the study, or the cost involved to acquire such data [2]. A study can be statistically more efficient than another if its estimates are more accurate (precise and valid), even if the studies involve the same number of subjects (and perhaps the same cost). “Cost” has a broad meaning here, including but not limited to the number of subjects involved in the study. Besides, sample size specific design strategies can enhance or impair the statistical efficiency of a study, depending on how the subjects are distributed in subgroups pertinent to the analysis.

### Definition of the population(s) to be sampled in cohort studies

In most ZKVP cohort studies to date (which follow the protocol for ZKVP studies recommended by the WHO [3]), when choosing groups for comparison (of exposed and unexposed subjects), there is an a priori identification of the source population (pregnant women, regardless of their exposure status) which is then sampled or included in its entirety (census). Subsequently, the group of pregnant women is classified according to exposure (presence or absence of ZKVP), respecting the exposed:unexposed ratio in the source population. Among the many advantages of this method, it allows a direct estimate of the risk of exposure to infection by the Zika virus (including both symptomatic and asymptomatic cases) in the general population of pregnant women (source population). However, this approach compromises the efficiency of a study considering the rare-exposure context, since the required minimum sample size may be larger than the sample size required in other strategies to achieve the same (or even better) statistical power. For example, let’s consider the following parameters: in a given population, 1.0% of pregnant women will be infected by the Zika virus (exposed group); among them, 5.0% will present the outcome of interest to the study (i.e., neurological changes in the conceptus), and 0.25% will present the outcome among the unexposed pregnant women. In total, it would require a minimum sample size of approximately 2,700 pregnant women to find a mere 27 exposed, and a total of eight outcomes (one among the exposed and seven among the unexposed mothers) to arrive at a statistical power of 59% and a type 1 error of 5% for crude analysis [4] (Table 1, example 1). Despite the large sample size, the small number of outcomes would greatly limit the efficiency of the study for stratified or multivariate analyses. It is clear that to better understand the natural history of ZKVP, more efficient studies are needed, including those supported by reliable data bases that allow testing for interactions and adjusting for confounding variables.

**Table 1:** Different sample strategies and characteristics to assess outcomes associated with Zika infection during pregnancy in analytical cohort studies

Characteristics	Sample of study population	Sample of unexposed pregnant women, and census of exposed population (from study population).	
	Example 1	Example 2	Example 3
Study population size of eligible pregnant women (Study population)	20.000	20.000	20.000
Sample size	2.700	800 (ratio 1:3)	1.200 (ratio 1:5)
Exposed (sample)	27 (1%)^a^		
Unexposed (sample)	2673		
Exposed (from population identified by surveillance systems)	–	200 (1%)^a^ exposed	200 (1%)^a^ exposed
Unexposed (from population identified by surveillance systems)	–	600–6 exposed (1%) = 594 unexposed	1.000–10 exposed (1%) = 990 unexposed
Ratio exposed:unexposed	1:99 (as the study population)	1:3 (defined by investigator)	1:5 (defined by investigator)
Expected cases among exposed (rounded numbers)	1 (5%)^a^	10 (5%)^a^	10 (5%)^a^
Expected cases among unexposed (round numbers)	7 (0.25%)^a^	1 (0.25%)^a^	2 (0.25%)^a^
Total expected cases (round numbers)	8	11	12
Type 1 error	0.05	0.05	0.05
Study Power^b^	59%	95%	97%

^a^Parameters: 1% of pregnant women will be infected by the Zika virus; 5 and 0.25% of infected and non-infected pregnant women will present the outcome, respectively

^b^StataCorp. *Stata Statistical Software: Release 10*. College Station, TX: StataCorp LP. 2007 [4]

Aware of this limitation, we propose that the sample selection be done from the a priori identification of groups of pregnant women exposed and unexposed to ZKVP. This method will allow for an oversampling (even 100%) of the pregnant women with ZKVP and a optimized sampling from the general population of pregnant women unexposed to ZKVP, saving resources in the unexposed group and improving the expected number of incident cases (outcomes) overall. In the context of non-association, a ratio of 1:1 between exposed and unexposed would be most efficient. Recognizing a priori that there is a known association and that exposure to ZKVP is a risk factor, the ratio should favor the group of unexposed mothers where fewer cases (outcomes) are expected. Thus, one could choose an optimized ratio for exposed and unexposed pregnant women of 1:3 or 1:4 or 1:5 [5] (Table 1, examples 2 and 3).

Considering the same parameters described above, a sample of only 1200 pregnant women (200 exposed and 1000 unexposed, 1:5 ratio) would give better results compared to those obtained in a sample more than two times as large, as in the strategy previously described. Approximately 12 outcome cases would be found, ten among exposed and two among unexposed pregnant women, ensuring a higher statistical power (97%) in crude analyses than in the previous example [4] (Table 1, example 3). Table 1 shows expected results for these two sampling strategies.

We acknowledge the difficulty of identifying populations of ZKVP-exposed and unexposed pregnant women and the biases inherent in the selection of subjects based on secondary data. However, many countries, including Brazil, possess consolidated health information systems and have adopted surveillance protocols targeting women suspected of having ZKVP or other vertically communicable exanthematous diseases. In such countries, the following procedures may be helpful to identify exposed and unexposed pregnant women: i) an a priori identification of symptomatic pregnant women with suspected ZKVP through routine surveillance notification and of peers in the prenatal care services without suspected ZKVP; ii) posteriorly, to carry out laboratory tests to confirm or rule out the presence of ZKVP in both groups, allowing to recompose the exposed and unexposed groups; iii) subsequently, to follow-up the unexposed group (including lab tests) of women for the detection of exposure if it occurs at any time during the remaining gestational period, and again to recompose the exposed and unexposed groups. As an additional gain, detection bias by surveillance protocol can be estimated from the results of tests conducted on samples collected from the pregnant women unsuspected of having been exposed to ZKVP.

We also recognize the limitation of the currently available commercial laboratory test to accurately diagnose Zika virus infection during pregnancy, mainly in the asymptomatic cases with probable low viremia or antibody response. New tests are being developed, including the MAC-Elisa IgG and IgM test developed by the CDC to differentiate dengue fever infection and Zika infection by running both antigens together [6]. However, there is a need to develop rapid diagnosis tests to support health care professionals more efficiently when assisting pregnant women at risk of ZKVP.

## Conclusion

We hope that this reflection will broaden the methodological debate on this subject. The sampling strategy we have proposed may improve the statistical power of studies that aim to evaluate the association between Zika infection during pregnancy and outcomes for the offspring, as well as those with similar objectives. Besides that, the harmonization of Zika virus research protocols is also desirable to obtain comparable data in order to allow evidence generation regarding very rare outcomes when individual study power will probably not be sufficient.
